# Efficacy and safety of Qinghua Zhixie Decoction against diarrhea-predominate irritable bowel syndrome: A protocol for a randomized controlled trial

**DOI:** 10.1097/MD.0000000000028895

**Published:** 2022-03-04

**Authors:** Lijiang Ji, Xiaoying Zhao, Yuyan Zhang, Ping Zhao, Rui Gong, Fang Li, Hua Huang

**Affiliations:** a Department of Anorectal Surgery, Changshu Hospital Affiliated to Nanjing University of Chinese Medicine, Changshu, Jiangsu, China.; b Tianjin University of Traditional Chinese Medicine, Tianjin, China.; c Department of Gastroenterology, Changshu Hospital Affiliated to Nanjing University of Chinese Medicine, Changshu, Jiangsu Province, China.

**Keywords:** Chinese herbal formula, complementary and alternative medicine, diarrhea, irritable bowel syndrome, quality of life, recurrence

## Abstract

**Background:**

Diarrhea-predominant irritable bowel syndrome (D-IBS) is the main subtypes of irritable bowel syndrome (IBS). In recent years, more than half of IBS patients have received complementary and alternative medicine. Traditional Chinese herbal formula is widely used in Asia, and clinical studies have also found that Chinese herbal formula could significantly improve abdominal pain and diarrhea. We plan to carry out a randomized, controlled, double blind, clinical studies to observe the clinical efficacy of Qinghua Zhixie decoction in the treatment of D-IBS.

**Methods:**

Four hundred sixty-four participants will be randomly assigned to the treatment group and control group. Patients in both groups would take medications and stimulations simultaneously. The outcomes of IBS symptom severity score, quality of life, psychological states, and recurrence rate will be recorded. Statistics will be analyzed with the SPSS 22.0.

**Conclusions:**

The findings of the study will identify the safety and efficacy of Qinghua Zhixie decoction in the treatment of D-IBS.

**Trial registration: OSF Registration number:**

DOI 10.17605/OSF.IO/C8MHW.

## 1 Introduction

Irritable bowel syndrome (IBS) is characterized by recurrent abdominal pain, abdominal discomfort, abdominal distension, changes in bowel habits, and abnormal stool.^[1]^ IBS is common to society, with a prevalence of 3% to 20% in the USA, and 5% to 10% in Asia, according to the epidemiological investigation.^[2,3]^ Diarrhea-predominant irritable bowel syndrome (D-IBS) is the main subtypes of IBS and accounts for 40% of all affected individuals.^[4]^ Patients with D-IBS typically suffer from abdominal pain associated with frequent loose stools, and acute diarrhea is a common symptom.^[5]^

The causes of D-IBS are still not clearly defined, and it is generally believed that enteric neuromuscular dysfunction, visceral hypersensitivity, alterations in the composition of fecal microbiota, inflammation, mental and psychological disorders, and dysfunction of the brain-gut axis are involved.^[6,7]^ D-IBS is easy to recur, and there is still no specific treatment against it. At present, individualized symptomatic treatments, including antidiarrheal, improving intestinal motility, antidepressant, supplemented by psychotherapy and diet conditioning can only temporarily relieve individual symptoms, and a large number of patients reportedly discontinued the medications because of dissatisfaction.^[8,9]^ Moreover, many side effects, including head-ache, dizziness, dry mouth, and insomnia, cardiovascular disorders, and ischemic colitis have been reported after long-term use of those medications.^[10]^ In recent years, complementary and alternative medicine (CAM) has become widely accepted in IBS patients, and more than half of D-IBS patients have received CAM.^[11,12]^

Traditional Chinese herbal formula (CHF) is a widely used CAM therapy in Asia, and clinical studies have also found that CHF could significantly improve abdominal pain and diarrhea against D-IBS patients.^[13–15]^ We have been committed to the clinical application of D-IBS with CHF of Qinghua Zhixie decoction (QZD) for a long time, and have achieved innovative effects. The formula consisted of Fengfei Cao (*Pteris multifida Poir*), Dijin Cao (*Elsholtzia Ciliata Hyland*), Bugu Zhi (*Psoralea corylifolia*), Huanglian (*Coptis chinensis*), Muxiang (*Radix Aucklandiae*), Paojiang (*Baked ginger*), Baishao (*Radix paeoniae alba*), Fangfeng (*Radix Saposhnikoviae*), Cangzhu (*Rhizoma atractylodis*), Baizhu (*Rhizoma Atractylodis Macrocephalae*), and Xianhe Cao (*Agrimonia pilosa*). In the formula, berberine, the main active ingredient of Coptis chinensis, can regulate the intestinal flora to improve abdominal pain and diarrhea symptoms in IBS,^[16,17]^ and the combination of Radix Saposhnikoviae, Rhizoma Atractylodis Macrocephalae, and Radix paeoniae alba can improve the imbalanced fecal microbiota, reduce visceral hypersensitivity, and regulate the secretion of brain-gut peptides in patients with D-IBS.^[18,19]^ Therefore, we plan to carry out a randomized, controlled, double blind, and prospective clinical studies to observe the clinical efficacy of QZD compared with pinaverium bromide (PB) in the treatment of D-IBS.

## 2 Method

### 2.1 Study design

The study is a single-center, double blinded, double doomy, randomized, controlled clinical trial. The study has been approved by the Ethics Committee of Changshu Hospital Affiliated to Nanjing University of Chinese Medicine, and the trial will be carried out in accordance with the Declaration of Helsinki. The clinical trial has been registered on open science framework on January 18, 2022 (Registration number: DOI 10.17605/OSF.IO/C8MHW). The protocol conforms to the Standard Protocol Recommendations for Interventional Trials (SPIRIT) 2013 statement,^[20]^ and the results will be reported according to the CONSORT statement extension for trials.^[21]^ The schedule of enrollment, interventions, and assessments is shown in Table 1.

**Table 1:** Schedule of enrollment, intervention, and assessments.

	Enrollment	Treatment					Follow up
Time point	Week 0	Week 1	Week 2	Week 4	Week 6	Week 8	Week 16
Enrollment							
Eligibility screen	√						
Informed consent	√						
Demographics	√						
History of disease	√						
Allocation	√						
Intervention							
QZD+PB-simulation		√	√	√	√	√	
PB+QZD-simulation		√	√	√	√	√	
Assessments							
IBS-SSS		√	√	√	√	√	√
IBS-QOL	√					√	√
HAMD scale	√					√	√
Recurrence rate							√
Adverse effects				√		√	

HAMD = Hamilton depression, IBS-QOL = irritable bowel syndrome-quality of life questionnaire, IBS-SSS = irritable bowel syndrome symptom severity score, PB = pinaverium bromide, QZD = Qinghua Zhixie decoction.

### 2.2 Participants

**Figure 1:**
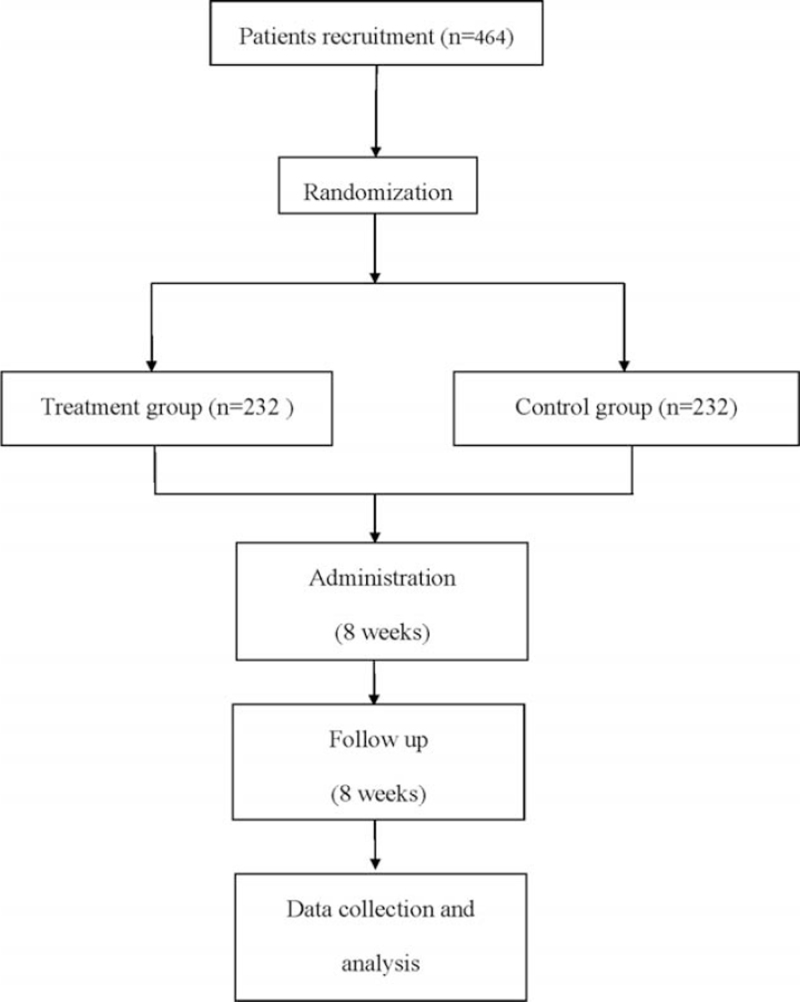
Flow diagram of this study.

Participants will be recruited from inpatients in Changshu Hospital Affiliated to Nanjing University of Chinese Medicine, and they will be randomly assigned to either the treatment group and control group by a 1:1 ratio (Fig. 1).

### 2.3 Inclusion criteria

(1) Patients meeting the diagnostic criteria for D-IBS;
(2) Patients aged from 18 to 70 years old;
(3) Patients without taking any drugs that would influence the results of this study at least 2 weeks before enrollment;
(4) Patients with written informed consent.

### 2.4 Exclusion criteria

(1) Patients with other gastrointestinal diseases, such as gastrointestinal tumors, anal diseases, intestinal obstruction, perforation, tumors, and anal diseases;
(2) Patients with a history of bowel surgery;
(3) Women in pregnancy, lactation, and menstruation;
(4) Patients taking non-steroidal anti-inflammatory drugs within 1 month that may affect visceral sensitivity;
(5) Patients with a history of allergies or allergic to Chinese herbs.

### 2.5 Dropout criteria

Patients will be dropped if they are with the following conditions:

(1) With severe adverse events (AEs);
(2) Unwilling to continue the study protocol.

### 2.6 Interventions

#### 2.6.1 Treatment group

Patients in the treatment group will take Chinese herbal formula QZD, which is composed of Fengfei Cao (*Pteris multifida Poir*) 30 g, Dijin Cao (*Elsholtzia Ciliata Hyland*) 15 g, Bugu Zhi (*Psoralea corylifolia*) 15 g, Huanglian (*Coptis chinensis*) 2 g, Muxiang (*Radix Aucklandiae*) 6 g, Paojiang (*Baked ginger*) 6 g, Baishao (*Radix paeoniae alba*) 15 g, Fangfeng (*Radix Saposhnikoviae*) 10 g, Cangzhu (*Rhizoma atractylodis*) 10 g, Baizhu (*Rhizoma Atractylodis Macrocephalae*) 15 g, and Xianhe Cao (*Agrimonia pilosa*) 12 g (the ingredients of each herb are provided in Supporting Material 1). The herbs are provided as herbal concentrate-granules in 1 bag and quality controlled by Tianjiang Pharmaceutical Co., Ltd. (Jiangyin, China). Patients will orally take 100 mL of the QZD twice a day and PB-simulation 50 mg 3 times a day continuously for 8 weeks.

#### 2.6.2 Control group

Patients in control group will take PB (50 mg, Abbott Products SAS, H20110220) 50 mg 3 times a day and QZD-simulation 100 mL twice a day continuously for 8 weeks.

### 2.7 Outcome variables

Patients will be asked to use a diary to record daily stools emissions, symptoms, frequency, and severity of AEs, and use of rescue medication.

#### 2.7.1 Primary outcome

Irritable bowel syndrome symptom severity score (IBS-SSS)^[22]^ is the primary outcome to record the degree and frequency of abdominal pain, abdominal distention, defecation satisfaction, and quality of life at weeks 0, 1, 2, 4, 6, 8, with 4 grades: remission (less than 75 points), mild (76–175 points), moderate (176–300 points), and severe (over 300 points).

#### 2.7.2 Secondary outcomes

(1) Quality of life: The IBS-quality of life questionnaire^[23]^ will be applied to to assess the patients’ quality of life with the following aspects: emotional state, psychological state, sleep, energy, daily activities, eating habits, social activities, major activities or work, and sex life, and a higher score reflects a lower quality of life. The questionnaire will be completed before and after treatment.
(2) Psychological states: The Hamilton depression scale^[24]^ will be used to evaluate the depressive states of patients during the clinical trial, and the Hamilton Anxiety scale^[25]^ for anxiety. The questionnaires will be completed before and after treatment.
(3) Recurrence rate: Patients will be followed up for another 8 weeks after the treatment, and the recurrence will be observed at week 16. If the IBS-SSS of a cured patient changes from remission to mild or a more severe state, he will be considered as recurrence.^[26]^ The recurrence rate = recurrent patients/total follow-up patients × 100%.

### 2.8 Safety assessment

The patient's vital signs, liver function, and renal function will be recorded at week 4 and week 8. All potential AEs will be accurately recorded at any time. If patients present with any discomfort, or AEs, they are instructed to inform the investigators. The physician will provide necessary treatment to alleviate any AEs.

### 2.9 Follow-up

After 8-week treatment, the patients will be followed-up for another 8 weeks to assess the recurrence rate.

### 2.10 Sample size

According to our preliminary experiment, QZD and PB are expected to reduce the IBS-SSS by 115 points and 105 points, respectively. Taking α = 0.05, β = 0.2, SD = 37, 212 cases will be needed in each group. Considering a loss to follow-up of 10%, 464 cases in total will be included.

### 2.11 Recruitment of participants

The study will be carried out at Proctology Department in Changshu Hospital Affiliated to Nanjing University of Chinese Medicine, and the patients will be recruited from inpatients via posters in the department.

### 2.12 Randomization and blinding

Patients will be randomly divided into treatment group (QZD group) and control group (PB group) according to a computer-generated randomization list after the recruitment in a 1:1 ratio, and the random number table will be sealed in a special envelope to be kept in a safe place until the study is complete. Due to the differences in appearance, taste, dosage, and administration methods between QZD and PB, a double-blinded and double-dummy design will be applied. The simulations are composed of amylum. Patients in both groups would take medications and stimulations simultaneously. The researchers, drug administrators, and patients are all unaware of the blind design, and the evaluators and the statisticians are not involved in the trial.

### 2.13 Discontinue and data monitoring

The supervisor will decide for discontinuing if more than 25% of the patients quit treatment due to AEs. Demographics, assessment, and cause of drop-outs will be recorded in Case report forms (CRFs), which will be submitted to the data management committee at the end of the study. All CRFs will be stored in a locked cabinet. The data management committee is independently chaired by the Statistics Teaching and Research Office of Changshu Hospital Affiliated to Nanjing University of Chinese Medicine and declares no conflict of interest. All CRFs will be preserved for at least 5 years after publication, and the access to the original data of readers and reviewers are available from the corresponding author on reasonable requests.

### 2.14 Statistical methods

Statistics will be analyzed with the SPSS 22.0 (SPSS Inc., Chicago, IL). Statistical testing is 2-sided and *P* < .05 is considered statistically significant. Efficacy outcome parameters will be analyzed on an intent to-treat basis, and the last observation carried forward rule will be applied to manage missing data. The results will be reported in descriptive statistics. According to the homogeneity, group *t* test for normally distributed data or Mann-Whitney *U* test for non-normally distributed data will be used to compare variables between groups.

### 2.15 Oversight and monitoring

The Office of Academic Research in our hospital will supervise and monitor the trial independently, and audit the trial twice per year. If it needs any changes to the protocol, we will first notify the sponsor. After making the revised protocol, we will update it in the registry site.

## 3 Discussion

In recent years, studies have found that the bacteria-gut-brain axis plays an important role in the development of D-IBS. The imbalance of intestinal flora could lead to disorders of the enteric nerve-immune-endocrine network, to stimulate the central nervous system to release brain-gut peptides, to increase visceral hypersensitivity.^[27–29]^ Therefore, regulating intestinal flora imbalance may be an effective way to treat D-IBS. Multiple researches demonstrate that CHF has been considered safe and effective for IBS,^[14,30–33]^ and CHF could also improve the intestinal flora imbalance, regulate the secretion of brain-gut peptide, improve intestinal digestion and absorption, inhibits intestinal movement, and reduce visceral hypersensitivity^[18,19,34]^ in patients with D-IBS.

QZD is a commonly used prescription in our group against D-IBS, and it is composed of Pteris multifida Poir, Elsholtzia Ciliata Hyland, Psoralea corylifolia, Coptis chinensis, Radix Aucklandiae, Baked ginger, Radix paeoniae alba, Radix Saposhnikoviae, Rhizoma atractylodis, Rhizoma Atractylodis Macrocephalae, Agrimonia pilosa. The main active ingredients, berberine, psoralenoside, quercetin, flavone, dehydrocostuslactone, and paeoniflorin in the formula could regulate the intestinal flora, reduce 5-hydroxytryptamine and vasoactive intestinal polypeptide to improve diarrhea.^[35–37]^ In this current randomized controlled clinical trial study, we will identify the safety and efficacy of QZD in the treatment of D-IBS.

However, there are still some limits in the design of this study. First, this study is a single-center study, and the source of cases is relatively single and limited, which may affect the conclusions of the study. Second, most of the outcome indicators used in this study are self-tested scales, and the results may result in subjective bias.

## Author contributions

Conception and design: Lijiang Ji and Fang Li. Administrative support: Lijiang Ji and Xiaoying Zhao. Provision of study materials or patients: Xiaoying Zhao and Ping Zhao. Collection and assembly of data: Rui Gong, Ping Zhao, and Yuyan Zhang. Data analysis and interpretation: Yuyan Zhang and Fang Li.

Funding support: Hua Huang.

Manuscript writing: All authors.

Final approval of manuscript: All authors.

**Conceptualization:** Lijiang Ji.

**Data curation:** Yuyan Zhang, Ping Zhao, Rui Gong.

**Funding acquisition:** Hua Huang.

**Project administration:** Xiaoying Zhao.

**Supervision:** Fang Li.
